# Effects of Nutrient Solution Application Rates on Yield, Quality, and Water–Fertilizer Use Efficiency on Greenhouse Tomatoes Using Grown-in Coir

**DOI:** 10.3390/plants13060893

**Published:** 2024-03-20

**Authors:** Shengxing Liu, Xiaoman Qiang, Hao Liu, Qisheng Han, Ping Yi, Huifeng Ning, Huanhuan Li, Chunting Wang, Xianbo Zhang

**Affiliations:** 1 School of Agricultural Sciences, Zhengzhou University, Zhengzhou 450001, China; liushengxing205@gs.zzu.edu.cn; 2 Key Laboratory of Crop Water Use and Regulation, Ministry of Agriculture and Rural Affairs, Farmland Irrigation Research Institute, Chinese Academy of Agricultural Sciences, Xinxiang 453002, China; liuhao03@caas.cn (H.L.); hanqisheng@caas.cn (Q.H.); 82101212136@caas.cn (P.Y.); ninghuifeng@caas.cn (H.N.); lihuanhuan@caas.cn (H.L.); 82101232118@caas.cn (C.W.); zhangxianbo@caas.cn (X.Z.)

**Keywords:** substrate-cultivated tomato, fertigation, yield, fruit quality, water and fertilizer use efficiency

## Abstract

The yield, quality, and water–fertilizer use efficiency of crops are important parameters for assessing rational water and fertilizer management. For an optimal water and fertilizer system with respect to the nutrient solution irrigation of greenhouse tomatoes using cultivation substrates, a two-year greenhouse cultivation experiment was conducted from 2022 to 2023. Three drip fertigation treatments (T1, T2, and T3) were implemented in the experiment, where nutrient solutions were supplied when the substrate’s water content reached 60%, 70%, and 80%. The frequency of nutrient solution applications is based on weighing coconut coir strips in the morning and evening at 7:00 to determine the daily water consumption of plants. Nutrient solutions were supplied when the substrate’s water content reached the lower limit, and the upper limit for nutrient supply was set at 100% of the substrate water content. The nutrient solution application was carried out multiple times throughout the day, avoiding the midday heat. The nutrient solution formula used was the soilless tomato cultivation formula from South China Agricultural University. The results show that plant height and the leaf area index rapidly increased in the early and middle stages, and later growth tended to stabilize; the daily transpiration of tomatoes increased with an increase in nutrient solution supply, and it was the greatest in the T3 treatment. Between the amount of nutrient solution application and the number of years, the yield increased with the increase of the amount of nutrient solution, showing T3 > T2 > T1. Although the average yield of the T2 treatment was slightly lower than that of the T3 treatment by 3.65%, the average irrigation water use efficiency, water use efficiency, and partial fertilizer productivity of the T2 treatment were significantly higher than those of the T3 treatment by 29.10%, 19.99%, and 28.89%, respectively (*p* < 0.05). Additionally, soluble solid, vitamin C, and soluble sugar contents and the sugar–acid ratio of tomatoes in the T2 treatment were greater than those in the other two treatments (*p* < 0.05). Using the TOPSIS (Technique for Order Preference by Similarity to an Ideal Solution) method, it was concluded that the nutrient solution application rate of 70% can significantly increase water and fertilizer use efficiency and markedly improve the nutritional and flavor quality of the fruit without a significant reduction in yield. This finding provides significant guidance for the high-yield, high-quality, and efficient production of coconut coir-based cultivated tomatoes in greenhouses.

## 1. Introduction

With the continuous increase in the world’s population and rapid social and economic development, the demand for food by humans is constantly increasing. However, with limited arable land resources, per capita land resources only comprise about one-fourth of the world average, and the per capita arable land area is less than one-third of the world’s total per capita arable land area [1,2]. In order to alleviate the contradiction between the scarcity of arable land resources in China and the increasing demand for food, it is particularly important to increase the yield and quality of non-arable lands, such as saline–alkali land, the Gobi Desert, and deserts. Soilless cultivation technology in facility cultivation is considered the optimal method for artificial fruit and vegetable cultivation that does not depend on space and the environment. The core technology of this method lies in the precise supply of irrigation and fertilization, offering various advantages such as independence from soil, standardized management, intelligent control, reduced manual labor, and the alleviation of environmental pollution [3,4]. However, water and fertilizer management is very strict during the process of facility cultivation, especially for leafy vegetables and melons. The control of water and fertilizer requirements for tomatoes, one of the major facility vegetables globally, has always been a hot research topic [5]. However, due to the fact that tomatoes are water-loving crops, the cultivation process requires a large amount of water and fertilizer. This often leads to problems such as insufficient or excessive water supply in the production of soilless cultivation of tomatoes, which severely hinders the sustainable development of the soilless cultivation of tomatoes [6]. Therefore, the development of precise management techniques for high-yield and high-quality soilless tomato cultivation facilities and the sustainable development of agricultural water resources depends on the efficient use of water and fertilizers.

Several studies have demonstrated the crucial role that plant growth and development and yield quality play in managing water and fertilizers in a soilless culture of tomato plants [7,8,9]. In their study on potted tomatoes, Li et al. [10] found that when the substrate moisture content stabilized at 76%, the yield and fresh matter accumulation were the highest. However, they also discovered that excessive substrate moisture content (88% moisture content) actually increased plant transpiration “inefficiency” and reduced crop water use efficiency. This shows that water and fertilizer supplies that are too high will result in a large stomatal opening of the plant, which is not conducive to photosynthesis. Within a certain range of irrigation, different levels of water and fertilizer applications have inconsistent effects on crop growth, yield, quality, and water and fertilizer use efficiency [11]. Research by Xia et al. [12] demonstrated that when the substrate moisture content was 50%, fruit quality and water use efficiency were significantly higher than in other treatments (substrate moisture content was 65%, 80%, and 95%), but yield and growth were lower (substrate moisture content of 80% and 95%). When the relative substrate moisture content was 80%, significant improvements were observed in leaf pigment content, water potential, osmotic potential, root vitality, and fruit yield, but there was a corresponding decrease in quality and water use efficiency. Patanè et al. [13] conducted a study using field-grown tomatoes "Red Star" as the experimental material and found that early water deficiencies significantly inhibited the accumulation of total dry biomass, resulting in a negative impact on yield but improved fruit quality, mostly in terms of total solids and total soluble solids. Despite the later water shortage, tomato yields did not decrease significantly, with an average water saving of 46.2% through irrigation.

Numerous scholars have engaged in discussions regarding the impact of substrate water and lower nutrient limits, as well as the regulations of water and nutrient control systems, on various indicators, such as crop yield, quality, and water use efficiency. However, these studies have not yet developed a comprehensive system that is applicable to the precise irrigation of nutrient solutions for tomato cultivation in controlled greenhouse conditions. Furthermore, there has been a lack of integrated evaluations on multiple objectives, including tomato yield, quality, and water–nutrient utilization efficiency. Therefore, this study conducted an integrated experiment with respect to drip irrigation and fertilization for tomato cultivation in facility substrates, with the aim of (i) analyzing the effects of different nutrient solution application rates on the growth and development of soilless-cultivated tomatoes in facilities; (ii) elucidating the impacts of different nutrient solution application rates on the yield, irrigation water use efficiency, and water and fertilizer use efficiency of soilless-cultivated tomatoes in facilities; (iii) investigating the effects of different nutrient solution application rates on the quality of soilless cultivated tomatoes in facilities; (iv) comprehensively evaluating tomato yield, quality, and water and fertilizer use efficiency using the TOPSIS method; and determining the precise irrigation indicators for substrate-cultivated tomatoes in order to provide technical reserves for the precise regulation and intelligent decision making of soilless cultivation technology.

## 2. Results

### 2.1. Effects of Different Nutrient Solution Application Rates on the Growth Indexes of Tomato Cultivated in Substrate

**Figure 1:**
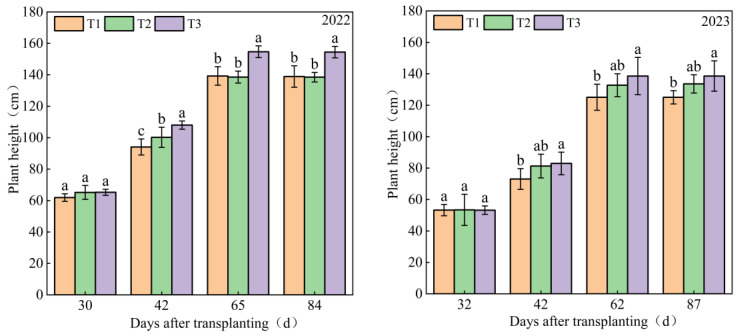

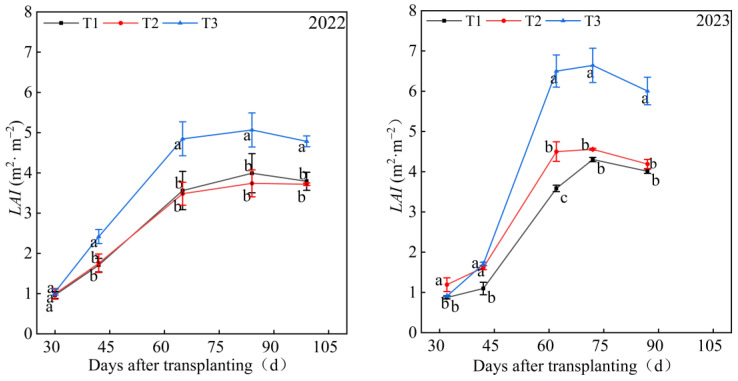
Effects of nutrient solution application rates on the plant height and leaf area index of tomatoes in substrate cultivation from 2022 to 2023 (note: lowercase letters indicate a difference between treatments at the 0.05 level using Tukey’s multiple range test. “T1” denotes the low nutrient application rate (substrate water-holding capacity of 60%), “T2” denotes the medium nutrient application rate (substrate water-holding capacity of 70%), and “T3” denotes the high nutrient application rate (substrate water-holding capacity of 80%); the holds for those below).

An essential indicator of a plant’s growth and development is its height. An important indicator of the growth status of plant groups is the leaf area index (*LAI*). The two indices typically have a strong correlation [14,15]. It can be observed in Figure 1 that the growth and change process of the plant’s height and the *LAI* of tomatoes in the two growing seasons comprises the following: The seedling stage trend is small, the flowering and fruit setting stage increases rapidly, and the mature picking stage tends to be gentle or decline. In the context of varying rates of nutrient solution application and average quarters, the elevation of the plant’s height and *LAI* values corresponded directly with the incremental supply of nutrient solutions. Notably, a high-nutrient-solution application rate (T3) exhibited the most notable outcomes, with a plant height of 146.52 cm and an LAI of 5.86 m^2^ m^−2^. Comparatively, the plant height and *LAI* values of the T3 treatment were significantly increased by 7.70%, 11.01%, and 41.13%, 41.18% (*p* < 0.05), respectively, compared with the medium-nutrient-solution application rate(T2) and low-nutrient-solution application rate(T1). Different nutrient solution application rates had significant effects on plant heights in both seasons. In 2023, the plant height of T3 was the highest at 87 days after transplanting, reaching 138.58 cm, while there was no significant difference between the T1 treatment, T2 treatment, and T3 treatment (*p* > 0.05). The comparison between plant heights and *LAI* reveals that during the mature phase, the changes in *LAI* values do not occur synchronously with the changes in plant height values. The observed trend of initially constant and then declining *LAI* values may be attributed to the prior topping of plants, where plant growth transitions from vegetative to reproductive growth. As the plants gradually age, their foliage becomes more closed, resulting in decreased light exposure to the lower leaves, which subsequently leads to dropping leaves.

### 2.2. Effects of Different Nutrient Solution Application Rates on the Daily Transpiration of Tomato Plants Cultivated in Substrates

**Figure 2:**
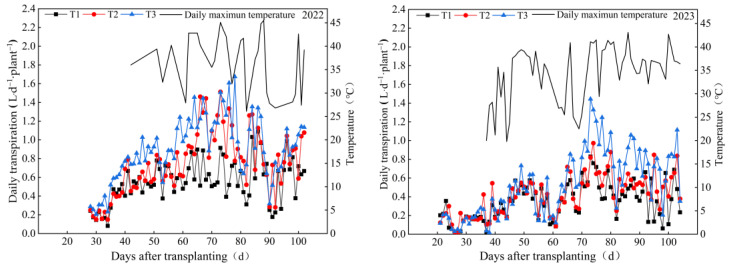
Changes in the daily transpiration of tomato plants and daily maximum temperature in a greenhouse under different nutrient solution rates from 2022 to 2023. The top line in the figure refers to the daily maximum temperature, and the three lines below it refer to the daily transpiration under the three fertigation treatments.

The daily transpiration of the two growing seasons was different with respect to the increase in nutrient solution supply, and the change in transpiration during different growth stages was not the same (Figure 2). During the seedling stage, the daily transpiration rates of the two tomato treatments did not differ significantly when the maximum daily temperatures were low. Subsequently, during the flowering and fruiting period, as temperatures gradually increased, the daily transpiration rates of the different treatments in 2023 showed the following trend: they were low at the beginning and the end and high in the middle. The maximum daily transpiration rates for all treatments occurred between 40 and 58 days after transplanting, and then they gradually decreased. This phenomenon can be attributed to a consecutive week of rainfall, which led to a decrease in maximum daily temperatures, reduced solar radiation, and a decrease in transpiration rates, resulting in a reduction in the daily transpiration rates during this stage. In terms of the average daily transpiration of the two seasons, compared with low-nutrient-solution application amount (T1) (0.476 L d^−1^ plant^−1^), the medium-nutrient-solution application amount (T2) (0.614 L d^−1^ plant^−1^) and high-nutrient-solution application amount (T3) (0.723 L d^−1^ plant^−1^), in addition to daily transpiration, increased by 28.99% and 17.75%, respectively; in terms of the average daily fertigation amount of the two growing seasons, compared with the T1 treatment (0.485 L d^−1^ plant^−1^), the daily fertigation amount of the T2 treatment (0.601 L d^−1^ plant^−1^) and T3 treatment (0.789 L d^−1^ plant^−1^) increased by 23.92% and 31.28%, respectively. Daily transpiration increased with an increase in nutrient solution supply.

### 2.3. Effects of Different Nutrient Solution Application Rates on Tomato Yield and Water and Fertilizer Utilization Rates under Substrate Cultivation

The single-fruit weight, the yield per plant, and the yield of tomatoes were all affected differently by different doses of the nutrient solution (Table 1). With an increase in the application of nutrient solutions, the single-fruit weight, yield per plant, and total yield of each treatment exhibited an upward trend: high-nutrient-solution application amount (T3) > medium-nutrient-solution application amount (T2) > low-nutrient-solution application amount (T1). Among them, the T3 treatment had the highest average values for the single-fruit weight, yield per plant, and total yield, which were 120.66 g, 1.67 kg, and 58,096.01 kg·ha^−1^, respectively. Compared with the T3 treatment, the T2 treatment decreased by 1.79%, 3.91%, and 3.65%, respectively, and there was no significant difference between the two (*p* > 0.05). The T1 treatment significantly reduced by 12.01%, 24.68%, and 25.56%. The results indicated that within two growing seasons, the T3 treatment exhibited the lowest average values for all indicators of irrigation water use efficiency, water use efficiency, and partial fertilizer productivity (22.71 kg m^−3^, 28.04 kg m^−3^, and 53.65 kg kg^−1^). Compared to the T3 treatment, the average values of the three indicators in the T1 treatment increased by 29.10%, 19.99%, and 28.89%, respectively, while in the T2 treatment, they significantly increased by 25.72%, 23.55%, and 26.01%, with significant differences between the two treatments (*p* < 0.05).

### 2.4. Effects of Different Nutrient Solution Application Rates on the Fruit Quality of Tomatoes

As shown in Table 2, the soluble solids and vitamin C of the two growing seasons exhibited a first increasing and then decreasing trend, both demonstrating the following: medium-nutrient-solution application amount (T2) > low-nutrient-solution application amount (T1) > high-nutrient-solution application amount (T3). The average values of the two growing seasons exhibited a significant increase of 14.15% and 21.68% for the T2 treatment compared to the T3 treatment, respectively (*p* < 0.05). In 2022, the T2 treatment exhibited a 34.42% higher soluble protein content compared to the T1 treatment and 16.30% higher content compared to the T3 treatment. In 2023, the soluble protein content increased gradually with the increase in nutrient solution application rate, demonstrating the following for the treatments: T3 > T2 > T1. There was no significant difference in the soluble protein content between the T3 and T2 treatments, with the T3 treatment being 7.52% higher than the T1 treatment (*p* > 0.05). The soluble sugar content and sugar–acid ratio of the two seasons initially increased and then decreased with an increase in nutrient solution application rates. The average value of these two indexes during the two seasons under the T2 treatment was 17.88% higher and 13.82% higher than that of the T3 treatment (*p* < 0.05), which is significant.

### 2.5. TOPSIS

The results of the three fertigation treatments (low-nutrient-solution application amount (T1), medium-nutrient-solution application amount (T2), and high-nutrient-solution application amount (T3)) from 2022 to 2023 were evaluated using the TOPSIS method based on various indicators, including the total tomato yield, irrigation water use efficiency, water use efficiency, partial fertilizer productivity, nutritional quality (total soluble solids, soluble protein, and vitamin C), and flavor quality (soluble sugar content, organic acidity, and sugar–acid ratio) (Table 3). The rankings and scores were assigned to each treatment for each year, with T1 < T3 < T2 in 2022 and T3 < T1 < T2 in 2023. A higher comprehensive evaluation coefficient (C*i*) indicates better treatment performance [16]. Based on the comprehensive indicators from these two years, both T2 treatments had the highest C*i* values, namely 0.738 and 0.355, suggesting optimal comprehensive benefits by considering yield, irrigation water use efficiency, water use efficiency, partial fertilizer productivity, nutritional quality, and flavor quality.

## 3. Discussion

Water and nutrients are key factors for the high-quality and high-yield cultivation of greenhouse crops, and the scientific and rational management of nutrient solutions is the core of substrate cultivation [17,18]. We found that the plant height and leaf area indexes exhibited an increasing trend with an increase in nutrient solution application rates from the flowering to the maturity stage. Among them, high-nutrient-solution application amounts (the matrix water-holding capacity is 80%) were significantly higher than those in other treatments (the matrix water-holding capacity is 60%, and the matrix water-holding capacity is 70%) (Figure 1), which is consistent with the research of Yang et al. [19]. This indicates that a sufficient nutrient supply can help plants maintain good growth statuses, better assist in the synthesis and transport of organic matter, and promote the vigorous growth of plants [20].

The quantity of nutrient solution supplies has a significant impact on the yield and water–fertilizer use efficiency of substrate-cultivated crops [21]. The yields maintained at a higher nutrient solution supply level were higher than other treatments, but the difference compared to the medium-nutrient-solution supply level was not significant. The water use efficiency and partial fertilizer productivity increased first and then decreased with an increase in fertigation amount (Table 1), which is consistent with the research results of Wu et al. [22] and Abdelghany et al. [23]. It indicated that the excessive vegetative growth of tomato plants under the long-term supply of 80 % nutrient solution caused excessive growth of plants, which did not play a positive role in the formation of yield. [24]. In addition, Cheng et al. [25] and Rasool et al. [26] argue that only when the amount of irrigation and fertilization is maintained at an appropriate level can higher yields be obtained and tomato quality be improved. Both excessive and insufficient water and fertilizer have inhibitory effects on tomato growth, which is consistent with the experimental results of this study. When the nutrient solution supply was reduced from the lower limit of 80% to 70%, the effect on tomato yields was not significant (Table 1), but its water use efficiency and partial fertilizer productivity were much lower than that of the medium-nutrient-solution supply. The reason is that when water and fertilizer inputs are excessive, the loss of invalid nutrients is increased. These findings indicate that the fertigation mode with the matrix water-holding capacity is 70%, which is more suitable for the coconut coir cultivation of tomatoes in terms of water and fertilizer requirements. It fully guarantees the generation and transport of organic matter and effectively maintains a balance between plant nutrition growth and reproductive growth, thereby ensuring tomato yield and improving water and fertilizer use efficiency.

During the nutrient solution supply process in substrate cultivation, water and fertilizers are inseparable as a whole. Water is the transport medium of fertilizers, and fertilizers can promote the absorption of water by plants. Tomato plants exhibit different abilities relative to absorbing and utilizing water and nutrients under varying levels of nutrient solution application, consequently resulting in variations in fruit quality. Under conditions of abundant nutrient supplies in the substrate solution without altering photosynthesis, the nutrients in the solution can alter the distribution of photosynthetic products among different organs, enhancing the fruit’s ability to absorb nutrients from the solution. This leads to fruit dilution and ultimately a decrease in the quality of tomato fruits [27]. When there is a deficiency in nutrient solutions in the medium, the demand for assimilates and water in tomato fruits during photosynthesis increases sharply. The water absorption rate of the plant’s root system is lower than the crop transpiration rate, resulting in the plant’s insufficient internal water content. As a result, the accumulation of water in the fruits decreases, leading to a decline in tomato fruit quality [28,29]. We found that the content of soluble solids, vitamin C, organic acids, and the sugar–acid ratio in tomato fruits exhibited an initial increase followed by a decreasing trend as the irrigation volume of the nutrient solution increased (Table 2). This finding slightly differs from the results of Wang et al. [30] and Li et al. [31]. The reason is that the planting methods of the two are different. The former comprises soil cultivation. This experiment uses substrate cultivation, and when the application amount of the nutrient solution reaches the threshold, continuing to increase the application amount of the nutrient solution does not significantly improve tomato quality [32]. Additionally, appropriate reductions in fertigation amounts can significantly improve the sugar–acid ratio of tomato fruits, enhance their taste, and increase economic benefits [33]. In the present study, when the matrix water-holding capacity was 70%, the soluble solid content, vitamin C content, soluble sugar content, and sugar–acid ratio were all significantly higher than in other treatments (*p* < 0.05). This indicates that the nutrient supply level promotes the absorption and utilization of nutrients by plants, stimulates plant growth, and thus enhances fruit quality. Therefore, considering aspects such as resource conservation and sustainable production, there is potential to reduce the nutrient supply level in substrate-cultivated tomatoes.

The ultimate goal of crop production is to achieve high yields, while the quality of crops is the desired outcome. The efficient utilization of water and fertilizer is a crucial component of effective agricultural water resource management. However, finding a balanced approach between these factors is challenging due to their complex relationship. Therefore, a comprehensive evaluation method that can be used to analyze these interrelationships is needed [34,35]. In this study, the TOPSIS method was used to evaluate the yield, irrigation water use efficiency, water use efficiency, partial fertilizer productivity, and overall quality of the three treatments in two growing seasons. The optimal nutrient solution application rate was estimated. Although there was slight inconsistency in the overall TOPSIS analysis over the two years, the matrix water-holding capacity of 70% exhibited the highest overall benefits (Table 3). Therefore, based on the results of this experimental study, the matrix water-holding capacity is 70%, and it not only increased the yield and improved water and fertilizer use efficiency but also maximized nutritional and flavor quality. This provides practical guidance for the actual production of tomatoes in a facility substrate cultivation system.

## 4. Materials and Methods

### 4.1. Overview of the Study Area

The study was conducted between April 2022 and July 2023 in the multi-field greenhouse of the Xinxiang Comprehensive Experimental Base (N 35°9′, E 113°47′, altitude 78.7 m), the Chinese Academy of Agricultural Sciences, which located in Qiliying City, Henan Province, Xinxiang County; the location had an average indoor air temperature of 23.5 °C, average air humidity of 77.54%, and average total radiation of 64.14 w m^2^, not involving wind speed and other factors. This region experiences warm, temperate continental monsoon weather. The average annual temperature is 14.1 °C, the average annual sunshine duration is 2398.8 h, the average annual precipitation is 548.3 mm, the average annual evaporation is 1908.7 mm, and the frost-free period is 200.05 days. The multi-span greenhouse occupies an area of around 2000 m^2^ and is located both in the north and the south. Lightweight, hot-dip galvanized steel was used to form the main frame, which was clad in 8 mm thick double glass. There are three greenhouses for testing, each measuring 28 m long and 9.6 m wide. There are 2 openings on the east and west sides and 3 openings in the middle. The greenhouses have a shoulder height of 5.5 m, a ridge direction ranging from the east to the west, a ridge height of 6.5 m, a wet curtain height of 1 m, and a fan height of 1.5 m. When the internal temperature rose above 35 °C and humidity exceeded 60%, the side windows and roof windows were closed, the external shading system was opened, and the cooling system was switched on. Natural ventilation at other times.

**Figure 3:**
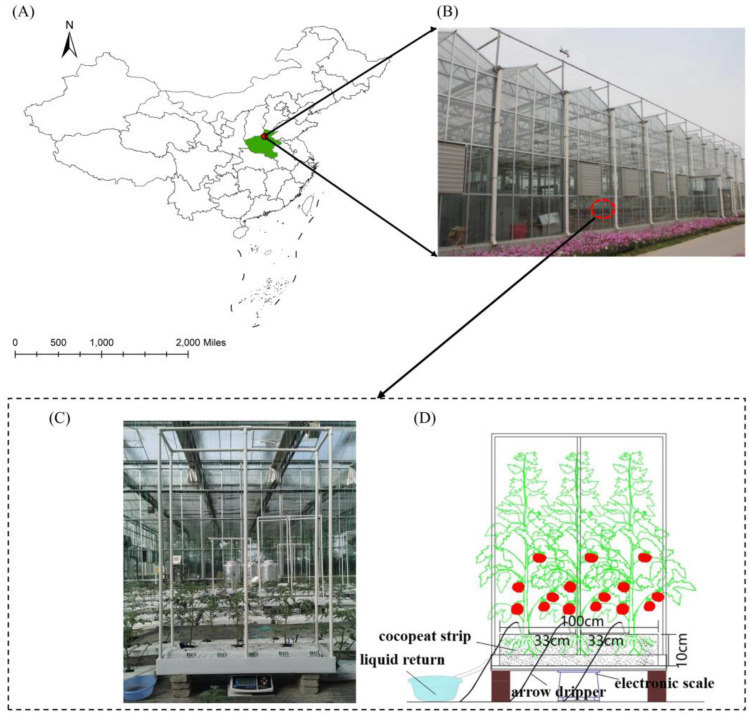
Schematic of the site and experiment carried out at the greenhouse test stations (**A**,**B**), including the cocopeat strips, liquid return, arrow dripper, and electronic scale (**C**,**D**).

The strawberry tomato variety tested is called “Jiamei” and has indeterminate growth potential. Four ears were left on each plant, and a topping treatment was applied to ensure the yield and quality of the tomatoes. The transplant was transplanted on 13 April 2022 and 27 March 2023, respectively. The harvest was finished on 23 July 2022 and 9 July 2023. A Dutch FORTECO Power coconut chaff was used as the substrate. The main component of the coconut chaff is a coconut shell. The surface of the coconut shell is negatively charged. Various cations are adsorbed on the surface of the coconut shell. Positive and negative ions attracted each other, and the composition was stable, which did not cause harm to the plants. Each substrate strip was compacted and filled with a plastic film wrapped on 6 sides according to the coarse–fine coconut bran ratio of 3:7, with a weight of 2.2 kg ± 0.02 kg and a bulk density of 0.0738 g cm^−3^. After soaking, the coconut chaff had a volume of 100 cm × 20 cm × 10 cm. All six sides were wrapped with plastic wrap except the top, which was divided into 5 cm× 5 cm square holes every 33 cm to accommodate tomato seedlings. Three tomatoes were planted in each 1 m long strip of the substrate. The greenhouse floor was paved with grass fabric, and there were drainage holes at the bottom. The experiment was carried out using the same row spacing cultivation method of coconut strips, with a row spacing of 100 cm and plant spacing of 33 cm. Each treatment had 6 rows, and each row had 22 matrix strips. Each treatment had three cultivation tanks with a volume of 110 cm × 30 cm × 10 cm, and each tank was placed with a coir (100 cm × 20 cm × 10 cm) and a cube frame (110 cm × 30 cm × 151 cm), with a total of 9 weighing matrix strips. The locations of the test station and the test setup in the greenhouse are shown in Figure 3.

### 4.2. Experimental Design

The experiment was conducted with three treatments based on the lower limit of the nutrient solution application rate: 60% of the substrate’s water-holding capacity (T1), 70% of the substrate’s water-holding capacity (T2), and 80% of the substrate’s water-holding capacity (T3). When the water content of each treatment reached the fertigation’s lower limit, the nutrient solution application rate was measured, with the upper limit of fertigation being 100% of the substrate’s water-holding capacity. The duration and frequency of the nutrient solution application rate for each treatment were adjusted based on the principle that the proportion of liquid return relative to the application amount was 25% to 30% [36]. Drip arrow fertigation was used as the fertigation method, with a drip head spacing of 33 cm and a drip head flow rate of 2 L h^−1^. The nutrient solution volume was controlled via a flow meter (accuracy of 0.001 m^3^). The experiment was arranged in a single-factor randomized block design with three replications for each treatment, resulting in a total of nine plots. The size of each plot was 22 m × 5.6 m, and the total number of plants per plot was 396. The specific nutrient solution volumes and application frequency are shown in Table 4.

Based on the previous research results reported by our project team on the drip fertigation system for tomato cultivation in a greenhouse, with reference to a water volume of approximately 200 mL (per plant)^−1^ during the seedling stage of soil-grown tomatoes, and in order to ensure the survival rate of the transplanted seedlings in this experiment, no fertigation treatment was applied to each treatment within 0–30 days after planting (specific timing depending on the plant growth), and the nutrient solution application rate was 198 mL (per plant)^−1^. The nutrient solution was applied twice a day, at 8:00 a.m. and 6:00 p.m., each time for 3 min. The fertigation treatment started at the end of the seedling stage (starting at 28 days after transplanting in 2022 and 21 days after transplanting in 2023). The nutrient solution application amount was determined based on the moisture content of the substrate in each treatment, with a duration of 5 min per drip fertigation at the end of the seedling stage and 8 min per drip fertigation from flowering to harvest. The drip fertigation frequency was calculated based on the nutrient solution application amount and duration, and fertigation was generally carried out in the morning and afternoon to avoid high temperatures, with the fertigation time controlled by an electromagnetic valve. The nutrient solution in the substrate was regularly monitored for electrical conductivity (EC) and pH using a portable conductivity meter (ZD-EC) and Bluelab pH pen, respectively, to ensure that the EC (1.5~2.8 mS cm^−1^) and pH (5.3~5.8) values of the nutrient solution in the substrate were within the appropriate range for soilless tomato cultivation [37]. To prevent salt accumulation in the substrate and avoid pipe blockage, the pipes were flushed every 2 days. The nutrient solution formula used was the one proposed by the Soilless Cultivation Technology Research Laboratory of South China Agricultural University for tomato cultivation (Table 5), with a dosage of 0.5 for the seedling stage (EC value is 1.2 mS cm^−1^) and 1 for each subsequent growth stage (EC value is 1.5 mS cm^−1^) [38]. The tomato growth period in 2022 was divided into three stages: a 42-day seedling stage (13 April to 24 May), a 36-day flowering and fruiting stage (25 May to 29 June), and a 24-day maturity and harvest stage (30 June to 23 July), totaling 102 days. The tomato growth period in 2023 was divided into three stages: a 37-day seedling stage (27 March to 3 May), a 28-day flowering and fruiting stage (4 May to 31 May), and a 39-day mature and harvest stage (1 June to 9 July), totaling 104 days. During the growth of tomato seedlings, there is a large demand for water and fertilizers. It is necessary to regularly observe the drying of the matrix strip and adjust the amount of fertigation in time. During the mature picking period, branches and leaves need to be pruned regularly. The agricultural practices in each treatment, such as pruning, spraying, and topping, were synchronized with local agricultural practices.

### 4.3. Test Observation Items and Methods

#### 4.3.1. Greenhouse Meteorological Factors

The automatic meteorological recording system at a height of 2 m in the middle of the greenhouse was used to monitor meteorological data in the greenhouse, mainly solar radiation (Rs), relative humidity (RH), and air temperature (Ta). Rs was measured using a pyranometer (LI200X, Campbell Scientific, Inc., Logan, UT, USA) with an accuracy of 0.2 kW (m^2^ (mV))^−1^. Ta and RH were measured using temperature and humidity sensors (CS215, Campbell Scientific, Inc., Logan, UT, USA), respectively. Prior to usage, all instruments underwent sensitivity testing. The temperature data were recorded at 11:00 every day. After each recording, the greenhouse microenvironment was adjusted according to the temperature of the day to ensure the normal growth of the plant.

#### 4.3.2. Water-Holding Capacity of the Substrate

Five dry coconut chaff strips without any damage were randomly selected, and the dry weight was weighed. Three 5 cm × 5 cm square holes were cut from the top every 33 cm, and water was added to the matrix at 1.5 L each time once every 15 min. Each coconut chaff strip was added with 20 L of water, and the excess water was removed after 12 h. On the second morning, excess water was removed, and the mass was weighed. Water-holding capacities were obtained by subtracting the mass twice, and finally, it was converted to an average water-holding capacity. The formula of the weight corresponding to the lower fertigation limit of each treatment is described as follows:

(1) X= M×N+W+m

where *X* is the corresponding weight when the water content of each treatment matrix reaches the lower limit, kg; *M* is the water-holding capacity of the substrate, kg; *N* is the ratio of the water content to the matrix water-holding capacity corresponding to the lower limit of each treatment (T1 is 60%, T2 is 70%, and T3 is 80%); *W* is the dry weight of the coconut chaff, kg; *m* is the weight of the cultivation trough and shelf, kg.

#### 4.3.3. Plant Water Consumption

Three coconut coir strips are placed on an electronic scale, which has a capacity of 30 kg and an error margin of less than 0.001 kg. The weight of the coconut coir strips is recorded at 7:00 am each morning (before fertigation), and the weight of the leachate is measured and recorded approximately 1 h after each fertigation. The crop water consumption formula [39] is described as follows:

(2) $$T_{V}=I_{i}+W_{i}-W_{i+1}-S$$

where *T_v_* is the daily water consumption of the plant, kg; *I_i_* is the irrigation amount of the day, kg; *W_i_* is the total weight of the plant together with the coconut chaff at 7:00 a.m. on the same day, kg; *W_i_*_+1_ is the total weight of the plant and the coir strips at 7:00 a.m. on the next day, kg; *S* is the total weight of the return liquid after fertigation on the same day, kg.

#### 4.3.4. Plant Growth Indicators

The main growth indicators for plants are measured in terms of plant height and leaf area. Starting approximately 15 days after transplanting, three representative plants were selected from each treatment at intervals of 10–15 days for measuring the plant’s height and leaf area. Each treatment was repeated 3 times. A ruler was used to measure the length (*a*) and width (*b*) of the leaves in centimeters (cm). The formula of crop leaf area index [40,41] is described as follows:

(3) $$LAI=\frac{\sum \left(a\times b\times 0.685\right)\times m\times 10^{-4}}{666.67}$$

where *LAI* is the leaf area index of the tomato, m^2^ m^−2^; *a* is the length of the leaf, cm; *b* is the width of the leaf, cm; *m* is the number of plants per acre, plant.

#### 4.3.5. Tomato Yield, Irrigation Use Efficiency, Water Use Efficiency, and Fertilizer Partial Productivity

For each plot, 12 disease-free and uniformly growing tomato plants were selected as the observation objects for yield. An electronic scale with an accuracy of 0.01 g was used to individually measure the yield of each plot and record the total number of fruits in each plot. The average value was taken as the average yield of the plot. Each treatment was replicated 3 times, and the final result was converted into the average yield per plant. Moreover, the single-fruit weight was converted. The formula for irrigation use efficiency (*IWUE*) [42] is described as follows:

(4) $$IWUE=\frac{Y_{a}}{I}$$

where *IWUE* is the irrigation use efficiency (kg m^−3^); *Y_a_* is the tomato yield (kg plant^−1^); *I* is the total irrigation amount of tomatoes during the whole growth period (m^3^ plant^−1^).

The calculation formula for water use efficiency (*WUE*) [43] is described as follows:

(5) $$WUE=\frac{Y_{a}}{ET}$$

where *WUE* is water use efficiency (kg m^−3^); *Y_a_* is the tomato yield (kg plant^−1^); *ET* is the total water consumption of tomato during the whole growth period (m^3^ plant^−1^).

The formula of the partial factor productivity (*PFP*) of fertilizers [44,45] is described as follows:

(6) $$PFP=\frac{Y}{F}$$

where *PFP* is the partial fertilizer productivity (kg kg^−1^); *Y* is the tomato yield (kg hm^−2^); *F* is the total mass of the N, P_2_O_5_, and K_2_O fertilizers (kg hm^−2^).

#### 4.3.6. Fruit Quality

After the fruits were ripe, the first fruit ear and the third fruit ear (flowering and fruit setting on the same day) were picked for quality determination, and the average value was taken as the final quality. Three replicates were taken in each plot, and six replicates with the same maturity were selected in each group. The samples were picked before 8:00–10:00 in the morning, and the fresh fruits were placed in sealed bags and sent to the laboratory. The fruits were washed with distilled water and wiped dry, and each fruit was ground and mixed with a mixer for quality measurement. The total soluble solid measurement was carried out using a handheld sugar meter (ATAGO, PR-32α, Tokyo, Japan). The content of vitamin C was determined using the 2,6-dichloroindophenol titrimetric method [46]. Organic acids were measured using the titration method [47]. Soluble proteins were determined using the Coomassie Brilliant Blue method [46]. The content of soluble sugars was determined using the Anthrone colorimetric method [48]. The sugar–acid ratio was determined by dividing the soluble sugar content of each sample by the organic acid content of each sample [49].

#### 4.3.7. Electrical Conductivity of Nutrient Solutions, Substrates, and Pondus Hydrogenii

The ZDS-PPM conductivity test pen was used to detect the prepared nutrient solution, and the detection time was about 15–60 s. After transplanting tomatoes, three substrate bars were randomly selected from each treatment, with each treatment being repeated three times. Portable conductivity meters were used for testing after approximately 15 to 30 days, with data detection taking around 10–15 s. At the same time, Bluelab pH meters were used to determine the substrate’s acidity and alkalinity (pH), with a detection time of approximately 3–5 s.

#### 4.3.8. Calculation of Optimal Nutrient Solution Applications Based on the TOPSIS Method for Tomato Composite Indexes

The approaching ideal solution sorting method, also known as the method of ideal and non-ideal solution distances, is a commonly used comprehensive distance evaluation method in multi-objective decision analysis. The basic idea is to define the ideal and non-ideal solutions of the decision problem, rank them based on the degree of proximity to the finite number of evaluation objects, and find the optimal solution. This paper uses the TOPSIS method to calculate the optimal nutrient solution application amount for tomatoes based on yields, irrigation water use efficiency, water use efficiency, partial fertilizer productivity, nutrient quality, and flavor quality, including the following 5 steps [50,51]:

Step1. The evaluation index matrix of yields, irrigation use efficiency, water use efficiency, partial fertilizer productivity, and comprehensive quality under different nutrient solution application rates was determined as follows:

(7) $$Y={\left(y_{ij}\right)}_{m\times n}=\left(\begin{array}{cccc} y_{11} & y_{12} & \cdots & y_{1m}\\ y_{21} & y_{22} & \cdots & y_{2m}\\ \vdots & \vdots & \ddots & \vdots \\ y_{n1} & y_{n2} & \cdots & y_{nm} \end{array}\right)\left(i=1,2,\cdots n;j=1,2\cdots ,m\right)$$

where *y_nm_* denotes the mth evaluation indicator for the nth treatment of the original data, *n* is 3 (number of treatments), and *m* is 10 (number of evaluation indicators).

Step2. The dimensionless evaluation matrix is described as follows:

(8) $$Z_{\mathrm{ij}}=\frac{y_{ij}}{\sqrt{\sum_{i=1}^{n}y_{ij}^{2}}}\left(i=1,2,\cdots n;j=1,2\cdots ,m\right)$$

where *Z_ij_* is the normalized *y_ij_*.

The dimensionless evaluation matrix is described as follows:

(9) $$Z={\left(z_{ij}\right)}_{m\times n}=\left(\begin{array}{cccc} z_{11} & z_{12} & \cdots & z_{1m}\\ z_{21} & z_{22} & \cdots & z_{2m}\\ \vdots & \vdots & \ddots & \vdots \\ z_{n1} & z_{n2} & \cdots & z_{nm} \end{array}\right)$$

Step3. The positive ideal solution and negative ideal solution are determined as follows:

(10) $$x^{+}=\left(x_{1}^{+},x_{2}^{+},\cdots x_{m}^{+}\right)$$

(11) $$x^{-}=\left(x_{1}^{-},x_{2}^{-},\cdots x_{m}^{-}\right)$$

Step4. The Euclidean distance is calculated from each evaluation object to the positive and negative ideal solutions.

The distance from each evaluation object to the positive ideal solution is described as follows:

(12) $$d_{i}^{+}=\sqrt{\sum_{j=1}^{m}{\left(x_{ij}-x_{j}^{+}\right)}^{2}}$$

The distance to the negative ideal solution is described as follows:

(13) $$d_{i}^{-}=\sqrt{\sum_{j=1}^{m}{\left(x_{ij}-x_{j}^{-}\right)}^{2}}$$

Step5. The comprehensive evaluation index of each evaluation target is as follows:

(14) $$Ci=\frac{d_{i}^{-}}{d_{i}^{-}+d_{i}^{+}}$$

where 0 < *Ci* < 1. When *Ci* is closer to 1, the tomato has the best comprehensive evaluation effect with respect to yield, irrigation utilization efficiency, water use efficiency, partial fertilizer productivity, nutritional quality, and flavor quality.

### 4.4. Statistical Analysis

Excel 2019 was used for data processing, Origin 2021 (Origin Lab, Northampton, MA, USA) was used for graphic design, and SPSS v.29 (IBM Crop., Armonk, NY, USA) was used for analysis of variance to test significance. Significant differences were assessed using one-way analysis of variance (one-way ANOVA), and Tukey’s multiple range test was carried out at a probability of (*p* ≤ 0.05). All results were presented as the mean ± standard error. The TOPSIS method was comprehensively calculated and analyzed by referring to the methods of Li et al. [52].

## 5. Conclusions

The results of this experiment showed that during the flowering and fruit setting period of the plant, 70% of the nutrient solution application rate promoted the growth of the plant, and there was no difference in the 80% yield of the nutrient solution application rate; moreover, the water and fertilizer utilization efficiency of 70% of the nutrient solution application rate was higher than that of the nutrient solution application rate of 80%. At the same time, it can significantly improve the nutritional quality and taste flavor of the fruit; the results of the TOPSIS method also showed that the yield, irrigation water use efficiency, water and fertilizer use efficiency, and quality index of tomatoes were the highest under the condition of a nutrient solution application of 70%. So, using water and fertilizer management is recommended when the application amount of the nutrient solution is 70%, especially in areas where water resources are easily insufficient, which can significantly improve irrigation water efficiency, water use efficiency, and partial fertilizer productivity without reducing yield. Therefore, the nutrient solution fertigation scheme can provide a reference for the high-yield, high-quality, and high-efficiency production of tomato substrate cultivation (coconut coir strands) in North China. Future research should combine the level of irrigation and fertilization at different growth stages to study the effects of different irrigation and fertilization processes on tomato physiology and production under soilless cultivation in greenhouses.

## Figures and Tables

**Table 1:** Effects of different nutrient solution application rates on tomato yields and water and fertilizer use efficiency from 2022 to 2023.

Years	Treatments	Single-Fruit Weight (g)	Yield Per Plant (kg)	Total Yield (kg ha^−1^)	Total Water Consumption (m^3^ plant^−1^)	Irrigation Water Utilization Efficiency (kg m^−3^)	Water utilization Efficiency (kg m^−3^)	Partial Fertilizer Productivity (kg kg^−1^)
2022	T1	109.40 ± 2.08 b	1.64 ± 0.03 b	49,329.01 ± 945.21 b	0.042	29.88 ± 0.57 a	39.13 ± 0.75 a	69.53 ± 1.33 a
	T2	125.51 ± 2.57 ab	1.97 ± 0.08 a	59,218.17 ± 2469.05 a	0.056	31.82 ± 1.33 a	35.23 ± 1.47 a	73.48 ± 3.06 a
	T3	129.15 ± 15.27 a	2.06 ± 0.26 a	61,736.60 ± 7872.63 a	0.068	23.92 ± 3.05 b	30.25 ± 3.86 b	55.41 ± 7.07 b
2023	T1	105.84 ± 3.54 b	1.01 ± 0.07 b	43,227.54 ± 3086.91 b	0.033	27.19 ± 1.94 a	30.40 ± 2.17 ab	65.66 ± 4.69 a
	T2	111.40 ± 3.25 a	1.23 ± 0.16 a	52,843.98 ± 6763.50 a	0.039	26.90 ± 3.44 a	31.89 ± 4.08 a	64.95 ± 8.31 a
	T3	112.16 ± 0.23 a	1.27 ± 0.04 a	54,455.42 ± 1904.96 a	0.049	21.49 ± 0.75 b	25.82 ± 0.90 b	51.89 ± 1.82 b

Data are expressed as “means ± SE”. Letters in lowercase represent significant differences at *p* < 0.05 using Tukey’s multiple range test among different treatments.

**Table 2:** Effects of different nutrient solution application rates on tomato quality from 2022 to 2023.

Years	Treatments	Total Soluble Solids (%)	Soluble Protein (mg g^−1^)	Vitamin C (mg kg^−1^)	Soluble Sugar Content (%)	Organic Acidity (%)	Sugar–Acid Ratio
2022	T1	5.18 ± 0.33 b	4.30 ± 0.63 b	188.80 ± 23.06 b	2.52 ± 0.20 b	0.43 ± 0.04 a	5.88 ± 0.41 c
	T2	5.73 ± 0.53 a	5.78 ± 1.47 a	209.70 ± 22.07 a	2.94 ± 0.32 a	0.34 ± 0.03 b	8.81 ± 0.97 a
	T3	5.09 ± 0.16 b	4.97 ± 0.47 b	174.59 ± 10.56 b	2.64 ± 0.14 b	0.34 ± 0.03 b	7.70 ± 0.62 b
2023	T1	5.16 ± 0.37 a	3.19 ± 0.49 a	154.61 ± 8.63 a	2.39 ± 0.14 a	0.53 ± 0.04 a	4.51 ± 0.21 ab
	T2	5.37 ± 0.18 a	3.41 ± 0.65 a	156.25 ± 10.26 a	2.60 ± 0.11 a	0.53 ± 0.01 a	4.88 ± 0.30 a
	T3	4.64 ± 0.12 b	3.43 ± 0.65 a	126.78 ± 10.03 b	2.09 ± 0.11 b	0.49 ± 0.03 a	4.31 ± 0.25 b

Data are expressed as “means ± SE”. The letters in lower case represent significant differences at *p* < 0.05 using Tukey’s multiple range test.

**Table 3:** Analysis of tomato yield, water and fertilizer use efficiency, and quality from 2022 to 2023 based on the TOPSIS method.

Years	Treatments	Normalized Decision-Making Matrix										D+	D−	C*i*	Rank
		Total Yield	Irrigation Water Use Efficiency	Water Use Efficiency	Fertilizer Partial Productivity	Total Soluble Solids	Soluble Protein	Vitamin C	Soluble Sugar Content	Organic Acidity	Sugar–Acid Ratio				
2022	T1	0.365	0.445	0.484	0.441	0.406	0.410	0.452	0.405	0.390	0.384	0.029	0.030	0.505	3
	T2	0.439	0.474	0.436	0.466	0.449	0.551	0.502	0.472	0.308	0.576	0.018	0.051	0.738	1
	T3	0.457	0.356	0.374	0.351	0.399	0.474	0.418	0.424	0.308	0.503	0.030	0.035	0.532	2
2023	T1	0.457	0.356	0.374	0.351	0.405	0.304	0.370	0.384	0.480	0.295	0.045	0.024	0.345	2
	T2	0.320	0.405	0.376	0.416	0.421	0.325	0.374	0.417	0.480	0.319	0.042	0.023	0.355	1
	T3	0.391	0.400	0.394	0.412	0.364	0.327	0.304	0.336	0.444	0.281	0.047	0.017	0.268	3

“D+”, the Euclidean distance of the ideal solution; “D−”, the Euclidean distance of the negative ideal solution; “C*i*”, the comprehensive benefit evaluation index.

**Table 4:** The amount and frequency of nutrient solution application at different growth stages.

Year		2022			2023		
	Treatments	T1	T2	T3	T1	T2	T3
Perinatal Periods							
Seedling stage	Application rate (L plant^−1^)	13.861	12.824	17.121	5.804	7.401	7.042
	Application frequency (times palnt^−1^)	83	76	103	56	66	63
Flowering stage	Application rate (L plant^−1^)	25.62	32.187	43.907	10.729	12.89	15.27
	Application frequency (times palnt^−1^)	97	122	166	41	49	58
Fruit maturation stage	Application rate (L plant^−1^)	15.087	16.977	24.667	20.55	25.532	36.795
	Application frequency (times palnt^−1^)	57	64	93	78	97	139
Whole growth stage	Application rate (L plant^−1^)	54.568	61.988	85.695	37.083	45.823	59.107
	Application frequency (times palnt^−1^)	237	262	362	175	212	260

Note: In the seedling stage, the nutrient solution application rate was performed for 3 min each time period, 5 min each time after fertigation treatment, and 8 min each time in the flowering and fruit setting stage and mature picking stage; the specific times were determined according to the actual situation.

**Table 5:** Formulation of tomato nutrient solutions at the South China Agricultural University.

Categorization	Substantive Name	Basic Proportion (mg L^−1^)
Liquid A	Ca(NO_3_)_2_·4H_2_O	590
Liquid B	KNO_3_	404
	KH_2_PO_4_	136
	MgSO_4_ 7H_2_O	246
	FeSO_4_ 7H_2_O	13.9
Liquid C	C_10_H_14_N_2_Na_2_O_8_	12.5
Liquid D	H_3_BO_3_	2.86
	MnSO_4_ H_2_O	1.54
	ZnSO_4_ 7H_2_O	0.22
	CuSO_4_ 5H_2_O	0.08
	(NH_4_)_6_Mo_7_O_24_ 4H_2_O	0.02

## Data Availability

The raw data supporting the conclusions of this article will be made available by the authors, without undue reservation. The data are not publicly available due to copyrights cannot be available.
